# Solving a shuttle mystery

**DOI:** 10.7554/eLife.49831

**Published:** 2019-08-08

**Authors:** Bridget Conley, Jeffrey Gralnick

**Affiliations:** 1 BioTechnology InstituteUniversity of MinnesotaMinneapolis and Saint PaulUnited States; 2 Department of Plant and Microbial BiologyUniversity of MinnesotaMinneapolis and Saint PaulUnited States

**Keywords:** *Shewanella oneidensis*, extracellular electron shuttle, abiotic reaction, ACNQ, anaerobic bacteria, None

## Abstract

*Shewanella oneidensis* bacteria use an abiotic reaction to help shuttle electrons outside of the cell.

**Related research article** Mevers E, Su L, Pishchany G, Baruch M, Cornejo J, Hobert E, Dimise E, Ajo-Franklin CM, Clardy J. 2019. An elusive electron shuttle from a facultative anaerobe. *eLife* **8**:e48054. doi: 10.7554/eLife.48054

Life is powered by ‘redox’ reactions: electrons are released from a source (oxidation) and then transferred from one molecule to another until they are captured by a terminal electron acceptor (reduction). Humans, for example, oxidize glucose and reduce oxygen. However, many places on our planet lack glucose and oxygen, so the microorganisms in these ecosystems must rely on other molecules for their redox reactions. In particular, they must find other compounds to act as terminal electron acceptors.

In 1988, it was reported that the bacterium known as *Shewanella oneidensis* was able to use minerals such as manganese and iron oxides as terminal electron acceptors (Myers and Nealson, 1988). Known as extracellular electron transfer, this metabolism is unique because manganese and iron oxide cannot enter the cell, so the electrons must go outside to meet their acceptor. A diverse range of microorganisms can transfer electrons outside of the cell, suggesting this form of metabolism may be widespread in many ecosystems (Koch and Harnisch, 2016). Transferring electrons extracellularly means these species can change the solubility of important metal nutrients, but also that these bacteria can be harnessed in a variety of technologies including bioremediation, generating electricity in microbial fuel cells, and biosynthesis of valuable chemicals (Kappler and Bryce, 2017; Kato, 2015).

The major components of the extracellular electron transfer pathway in *S. oneidensis* have already been described: a donor compound gives electrons, which are then transferred to molecules such as menaquinone and c-type cytochromes, before they are delivered to the extracellular electron acceptor (Shi et al., 2016). Yet, how does an electron cross membrane barriers so it can get out of a bacterium and reach an extracellular acceptor? Electrons can be directly captured by the acceptor when in contact with electron-carrying proteins on the surface of the bacterium, but *Shewanella* species also secrete flavin molecules that can shuttle electrons to external compounds (Brutinel and Gralnick, 2012).

The first evidence showcasing that *S. oneidensis* may produce extracellular electron shuttles came from a mutant that was unable to synthetize a molecule known as menaquinone: this strain could not reduce a compound called AQDS unless it was physically close to wild-type cells (Newman and Kolter, 2000; Figure 1A). Analyzing the medium in which the wild-type bacteria were growing revealed the presence of a quinone-like molecule that could be ‘borrowed’ by the mutant bacteria. The role of this unidentified molecule has been the subject of debate: is it shuttling electrons to acceptors outside of the bacteria, or is it an intermediate in menaquinone synthesis that can fix the defect in mutant *S. oneidensis*? Now, in eLife, and nearly twenty years after its initial description, Jon Clardy and colleagues – including Emily Mevers and Lin Su as joint first authors – report that they have identified this molecule as ACNQ (2-amino-3-carboxyl-1,4-napthoquinone), a soluble analogue of menaquinone that can work as an electron shuttle (Mevers et al., 2019).

**Figure 1.**
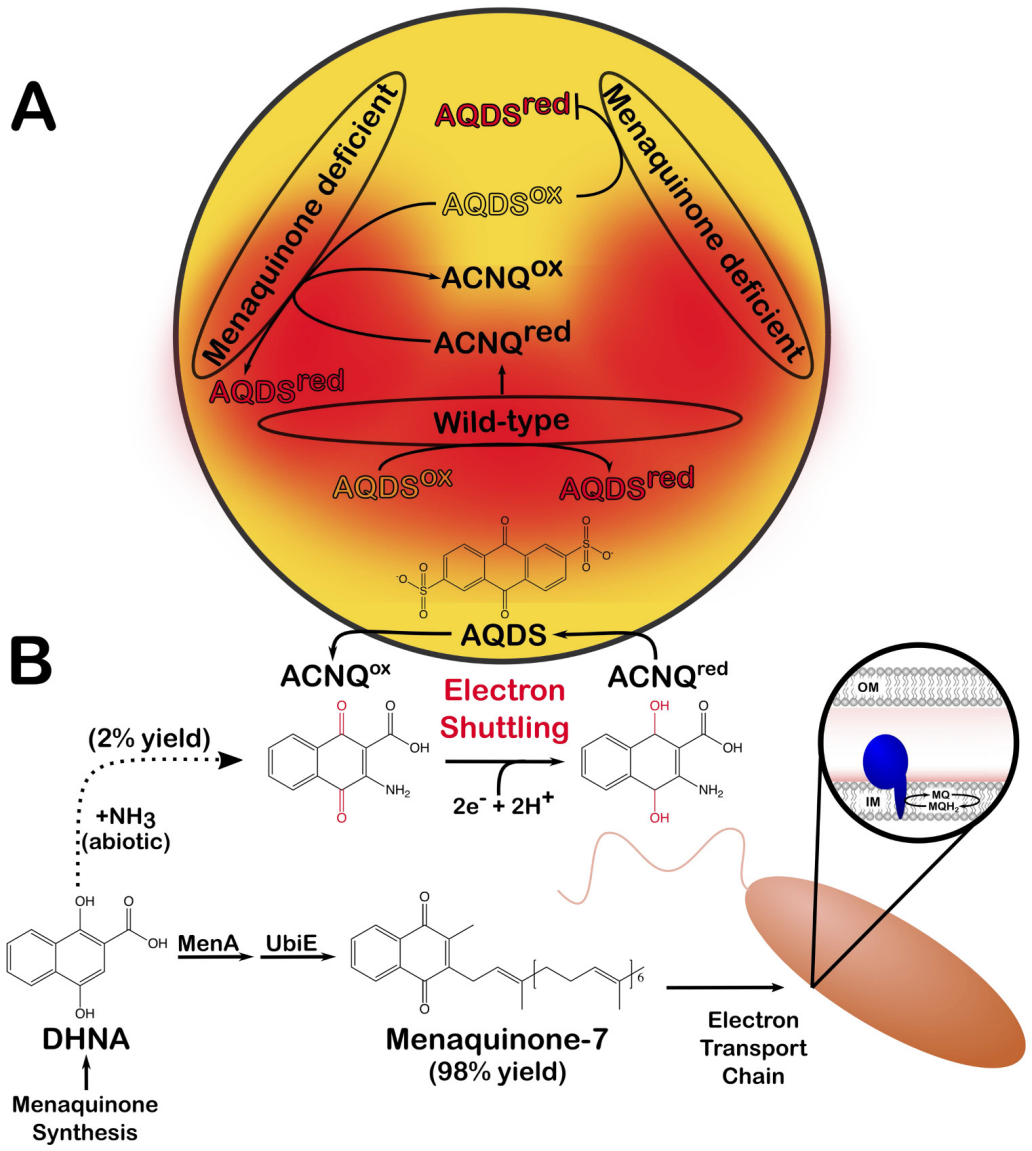
An abiotic reaction produces ACNQ, a molecule that serves as an electron shuttle in *Shewanella oneidensis.* (**A**) Redox reactions take place when electrons released from a molecule (oxidation) are accepted by another compound (reduction). Wild-type *S. oneidensis* can reduce AQDS, a molecule present in the environment (reduced AQDS is shown in red and oxidized AQDS in yellow); they also produce the newly identified compound called ACNQ, which shuttles electrons from the cells into the extracellular environment. When grown alone, mutant *S. oneidensis* bacteria that cannot produce menaquinone fail to reduce AQDS (upper right); however, when they are grown close to a wild-type colony, they can use the ACNQ molecules present in the milieu to complete the redox reaction (lower left). This diagram summarizes the AQDS reduction assays performed by Newman and Kolter as well as Mevers et al. (**B**) The work by Mevers et al. reveals how *S. oneidensis* can produce ACNQ. The enzymes MenA and UbiE convert the molecular precursor DHNA into menaquinone-7 (MQ), its dominant product. Menaquinone is found in the inner membrane (IM) of the bacterium, where it serves as a lipid-soluble electron carrier in the electron transport chain (blue structure in right inset). About 2% of the DHNA pool can also chemically react with an ammonia source (NH_3_^+^) to form ACNQ. AQDS: anthraquinone-2,6-disulfonate; ACNQ: 2-amino-3-carboxyl-1,4-napthoquinone; DHNA: 1,4-dihydroxy-2-naphthoic acid.

The team, which is based at Harvard Medical School, Lawrence Berkeley National Laboratory and Southeast University, confirmed that *S. oneidensis* is capable of producing ACNQ in culture, but at concentrations ten times higher than those observed in other microorganisms. Mevers et al. then focused on how this molecule is produced. Analysis of mutants defective in menaquinone synthesis suggested it is created from a molecule called DHNA, by a reaction that should be catalyzed via a transaminase. However, genetic analysis of *S. oneidensis* and *E. coli* did not yield any candidate enzymes that could produce ACNQ. This led to the intriguing hypothesis that ACNQ is synthetized from DHNA without the help of enzymes (Mevers et al., 2019). And indeed, Mevers et al. showed that, in a sterile medium, DHNA and an ammonia source could react to form ACNQ, demonstrating that the molecule could be created through an abiotic mechanism (Figure 1B).

Mevers et al. also explored whether ACNQ acts as an electron shuttle or as a menaquinone synthesis intermediate in *S. oneidensis*, as was debated previously (Newman and Kolter, 2000; Myers and Myers, 2004). Adding purified ACNQ to an *S. oneidensis* menaquinone mutant did not replenish the menaquinone pool, but it did restore the ability to reduce AQDS. A large dose of 1μM of ACNQ increased the rate at which the bacteria could transfer electrons to an electrode, but that mechanism did not require the cytochromes usually involved in extracellular electron transfer. These data support the hypothesis that ACNQ can work as an electron shuttle at micromolar concentrations in *S. oneidensis*. However, this activity may involve redox reactions that occur inside the cell (Yamazaki et al., 2002b) rather than the cytochrome-mediated mechanisms that are already known to participate in extracellular electron transfer (Shi et al., 2016).

The biological relevance of ACNQ remains an open question. In certain organisms, it can act as an electron sink and accept electrons, shifting the redox balance of the cell and directing the metabolism towards fermentation end products which are more energetically favorable (Freguia et al., 2009; Yamazaki et al., 2002a; Yamazaki et al., 2002b).

A variety of organisms that do not transfer their electrons outside still produce low levels of ACNQ, which may suggest other roles for the compound. For instance, it may be involved in cell-to-cell communication as well as in unconventional cellular warfare where redox balance is disrupted, or act as a nutrient for surrounding organisms. Regardless of the biological role(s) of ACNQ, Mevers et al. highlight that abiotic processes can add to gene-encoded activities to enhance metabolic diversity.
